# Factors Affecting the Implementation of Corporate Social Responsibility in the Health Technology Industry in Greece

**DOI:** 10.7759/cureus.39946

**Published:** 2023-06-04

**Authors:** Margarita Liopa, Mary Geitona, Dimitra Latsou

**Affiliations:** 1 Department of Social and Educational Policy, University of Peloponnese, Corinth, GRC; 2 Department of Public Administration, University of Neapolis, Pafos, CYP

**Keywords:** greece, health technology industry, medical equipment, biomedical products, pharmaceuticals, corporate social responsibility (csr)

## Abstract

Background: Corporate social responsibility (CSR) is an evolving business strategy worldwide, focusing on the sustainability of the enterprise and the provision of multiple benefits to the societies and economies.

Objective: The aim of this paper was to explore the encouraging and deterrent factors for the implementation of CSR actions in companies specializing in pharmaceutical and biomedical products as well as in medical equipment in Greece.

Methods: A cross-sectional study was conducted (April to June 2021) in member-companies of the Hellenic Association of Pharmaceutical Companies, the Panhellenic Association of Pharmaceutical Industry and the Association of Health-Research and Biotechnology Industry. Data collection was carried out via an anonymous, self-administered questionnaire. Descriptive and inferential statistical analyses were performed, using SPSS version 25 (IBM Corp., Armonk, NY, USA). The significance level was set at p≤0.05.

Results: One hundred twelve questionnaires were distributed, out of which 87 were returned (response rate 77.7%). 81.1% of companies included CSR in their annual strategy, while only 32.4% of them follow the Global Reporting Initiative standards. The majority (62.2%) disposes ≤€100.000 from their annual turnover for CSR actions. The contribution to society and the ethical commitment of the enterprise are stated as the main encouraging factors for CSR, while bureaucracy and the lack of incentives as deterrents. Pharmaceutical companies reported social acceptance as the major CSR enabler compared to other companies (p=0.034), while companies specializing only in medical equipment/biotechnology mentioned industry competition (p=0.003). Bureaucracy has been revealed as the major disincentive for all participating companies. Corporate advertising is found as an important encouraging factor for the adoption of CSR for the international companies compared to the national ones (p=0.023). Moreover, 97.3% stated that the government should reward socially responsible companies by increasing financial incentives.

Conclusion: The health technology industry in Greece implements CSR actions. The company's contribution to society and its ethical commitment are important encouraging factors for CSR, while bureaucracy and lack of government incentives are the main deterrents. The reward of socially sensitive companies by the government would provide significant entrepreneurial and societal benefits, supporting the overall Greek economy.

## Introduction

Corporate social responsibility (CSR) is an emerging and constantly evolving managerial and marketing strategy initiated in companies worldwide. CSR targets ensure the sustainable development of the company while providing economic, social and environmental benefits to all stakeholders [1]. More specifically, it incorporates a wide range of actions related to philanthropy, volunteering, ethical working practices, education, environment and other areas, and has a significant impact on both the company itself and society [2-6]. Companies that demonstrate high levels of voluntary commitment and responsibility are rewarded through recognition by the governments, the stakeholders and the society [7,8]. However, during the past few years, companies' voluntary commitment has been improving and efforts are being made to create an internationally standardised framework for CSR. Efforts related to the rising of consumers’ expectations, regulators’ emphasis on the importance of CSR, and on the companies’ benefits, such as reputation, image and long-term sustainability [4,8]. In this context, the Global Reporting Initiative (GRI) has created guidelines and standards for companies to assess and report their impact on critical sustainability issues [9]. However, this process is not compulsory for the companies.

The cultural values of the company are a decisive factor for the adoption or not of CSR initiatives and often the organizational structure of the company has to undergo significant changes. Brand reputation, social acceptance, attracting new customers and the ethical commitment of the company are some of the encouraging factors for companies to undertake CSR actions [10-12]. However, there are significant obstacles to CSR uptake, including financial, lack of government support, bureaucracy and poor communication with stakeholders [10,13,14]. Identifying the factors that may positively or negatively affect the implementation of CSR actions is crucial [13,15-18].

CSR constitutes an essential component of a company’s strategy, targeting to achieve a balance between profitability and volunteering [19,20-24]. The factors influencing the implementation of CSR actions are similar for companies specialised in health technology. Specifically, these companies are frequently engaged in CSR because of their considerable influence on society's health and well-being. Health technology companies seek to implement CSR initiatives in order to build a strong corporate reputation, improve their relationship with stakeholders, attract and retain employees and acquire a competitive edge over rival companies [25,26]. Furthermore, they disclose their financial, social and environmental actions and risks in order to improve transparency and public opinion. Both are essential components of their broader approach to incorporating CSR [27].

In recent years, Greek entrepreneurship has been experiencing intense socio-economic distortions due to the economic and pandemic crises [28]. The health technology industry has been revealed as an important driver in strengthening the Greek economy and a driving force for investment in research and development (R&D). Moreover, during both crises, companies in Greece demonstrated high sense of responsibility through donations of pharmaceuticals, medical equipment and diagnostic tests to the National Health System (NHS), public health campaigns as well as supporting patients and other vulnerable groups [29-33]. In this context, the objective of this study was to investigate the factors affecting the operation of CSR in Greek companies specialising in pharmaceutical and biomedical products as well as in medical equipment.

## Materials and methods

Study design and sample selection

A cross-sectional study was conducted at the member-companies of the Hellenic Association of Pharmaceutical Companies (SFEE), the Panhellenic Association of Pharmaceutical Industry (PEF), and the Association of Health-Research and Biotechnology Industry (SEIV), from April to June 2021.

Stratified sampling was the methodology applied and data collection was carried out through a self-administered questionnaire. Companies specializing in pharmaceutical and biomedical products as well as in medical equipment were defined as strata. The questionnaire was sent via email to the Chef Executive Officers (CEOs) of the 112 multinational and national companies which met the following inclusion criteria: a) companies specializing in pharmaceuticals, biotechnological products and medical equipment and b) active business companies. The exclusion criteria were: a) subsidiaries companies b) companies producing skin care products, nutritional supplements, baby food and similar products and c) companies operating as distributors of health technology products. Companies being members in more than one of the abovementioned associations filled out only one questionnaire in order to avoid participants’ duplication.

Study instrument

An anonymous and self-completion questionnaire was developed based on a review of the international and Greek relevant literature [34,35]. In order to assess the validity of the content, clarity and completeness of the tool [36], a pilot study was carried out on a convenience sample of five companies from the target population. The questionnaire consisted of 34 questions on a) the socio-demographic, occupational characteristics of the sample and the CSR activities, b) the participants’ opinion on the factors affecting (positively and negatively) the implementation of CSR and c) issues related with government support to CSR sensitive companies. Moreover, the questionnaire included the following types of responses: multiple choice; ranking questions (4-point Likert scale), where 1 corresponds to “very important” and 4 to “not important”; and binary (Yes/No).

Statistical analysis

Mean values and standard deviation (SD) were used for the description of quantitative variables. Absolute (n) and relative (%) frequencies were used for the description of qualitative variables. The variables were binary or ordinal, so non-parametric tests were performed. The statistical significance of difference between two independent groups was assessed by using the Mann-Whitney test and among three groups using the Kruskal-Wallis test. Spearman's correlation coefficient was applied for the relationship between the encouraging and deterrent factors for CSR implementation. The significance level was set at p≤0.05. Statistical Package for Social Sciences (SPSS) version 25 (IBM Corp., Armonk, NY, USA) was used for statistical analysis.

## Results

For the purpose of the survey, 112 questionnaires were distributed, out of which 87 were returned (response rate 77.7%). In addition, 13 were excluded from the analysis as less than 50% were completed, thus, 74 companies constituted the final sample. In Table 1, the socio-demographic and occupational characteristics of the sample are presented. Most of the participating companies belonged to multinational operating companies in Greece (62.2%), 40.5% operated in pharmaceuticals and 45.9% had an annual turnover of ≥40.000.000€. The majority of the respondents were CEOs holding a postgraduate degree.

**Table 1:** Sample characteristics

	Number	Percentage
Respondents’ characteristics		
Legal status of Company		
Multinational operating company in Greece	46	62.2
National company	28	37.8
Company’s field of operation		
Pharmaceutical products	30	40.5
Medical equipment & Biomedical products	24	32.4
Companies specialising in both categories	20	27.1
Annual company turnover		
≤ 2.000.000€	10	13.5
2.000.001€ - 10.000.000€	8	10.8
10.000.001€ - 20.000.000€	14	18.9
20.000.001€ - 40.000.000€	8	10.8
≥ 40.000.000€	34	45.9
Working Department		
General Directorate (CEOs)	40	54
Financial and Commercial (managers)	14	18.9
Regulatory and Communication (managers)	10	13.5
Human resources (HR) (managers)	10	13.5
Educational level		
Bachelor degree	18	24.3
MSc/ PhD	56	75.7

**Figure 1:**
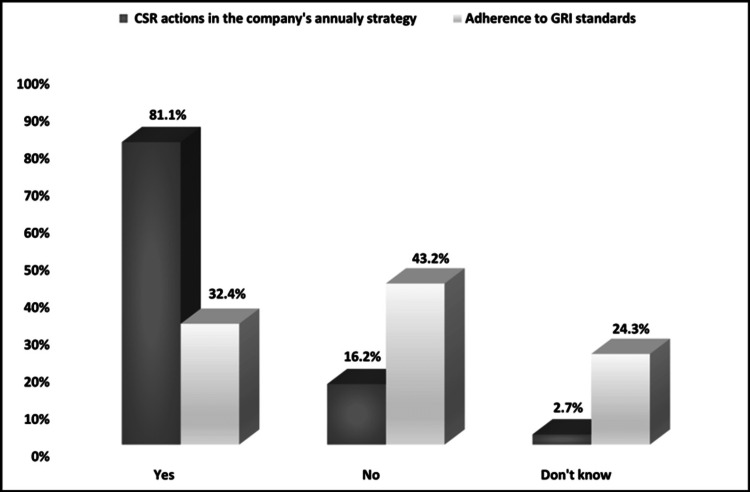
Annual corporate social responsibility (CSR) strategy and Global Reporting Initiative (GRI) standards

Moreover, 89.2% of the respondents stated that their company implements CSR activities, out of which 42.8% have more than 10 years of relevant experience. As shown in Figure 1, the majority (81.1%) incorporate CSR into the company’s annual strategy, but only 32.4% follow the standards of the Global Reporting Initiative (GRI). Also, 62.2% of companies dispose ≤€100.000 from their annual turnover for CSR actions and the rest (37.8%) more than €100.000. As far as the prioritization of CSR activities, healthcare and public health hold the priority (37.7%), followed by society (27.2%), environment (19.9%), market (8.6%) and human resources (6.6%).

Regarding the drivers for the implementation of CSR actions, the contribution to society was recorded as the most important reason, while the direct/indirect economic benefits as less significant (Table 2). As far as the deterrents are concerned, bureaucracy was mentioned as the most important factor, whilst unaware workers as the least important.

**Table 2:** Factors affecting corporate social responsibility (CSR) implementation 1 = very important; 4 = less important

	Mean (SD)
Encouraging factors	
Contribution to society	1.51 (1.04)
Ethical commitment of the enterprise	1.78 (1.08)
Transparency of corporate activities and transactions	1.92 (1.06)
Social acceptance	1.95 (1.05)
Corporate advertising	2.08 (1.03)
Working climate	2.08 (1.00)
Encouragement of customers and partners	2.22 (1.02)
Industry competition/ comparative advantage	2.22 (0.85)
Direct/ indirect economic benefits	2.89 (0.99)
Deterrent factors	
Bureaucracy	1.95 (1.10)
Lack of government incentives	2.05 (1.07)
Lack of regulatory framework	2.08 (1.08)
Financial cost	2.11 (0.80)
Unaware citizens	2.43 (0.76)
Unaware workers	2.59 (1.03)

As shown in Table 3, the results of the correlation analysis between the encouraging and deterrents factors, according to the opinions of the respondents: as social acceptance increases as an encouraging factor, bureaucracy, lack of regulatory framework, government incentives and unaware workers decrease as deterrents factors; as industry competition/comparative advantage and direct/indirect economic benefits increase as encouraging factors, the lack of regulatory framework, lack of government incentives and unaware workers also increase as deterrents; as corporate advertising increases as an encouraging factor, the financial cost increases and unaware workers decrease as deterrents; as the contribution to the society increases as an encouraging factor, the unaware workers decrease as a deterrent factor; as the motivation of customers and partners, the working climate and the ethical commitment of the company are increasing as encouraging factors, the unaware citizens are also increasing, as a deterrent.

**Table 3:** Correlation analysis among encouraging and deterrent factors for corporate social responsibility (CSR) * Correlation is significant at the 0.05 level (2-tailed). ** Correlation is significant at the 0.01 level (2-tailed).

Encouraging factors	Deterrent factors					
	Bureaucracy	Lack of regulatory framework	Lack of government incentives	Financial cost	Unaware workers	Unaware citizens
Social acceptance	-.279^*^	-.328^**^	-.232^*^		-.414^**^	
Industry competition/ comparative advantage		.325^**^	.357^**^			
Direct/indirect economic benefits		.279^*^	.298^*^		.428^**^	
Corporate advertising				.286^*^	-.340^**^	
Contribution to society					-.526^**^	
Motivation of customers and partners						.343^**^
Working climate						.256^*^
Ethical commitment of the enterprise						.380^**^

Additionally, regarding the field of companies’ operation, pharmaceutical companies mentioned that corporate advertising is the main encouraging factor for CSR actions, in contrast to the medical equipment and biotechnology companies which mentioned industry competition/comparative advantage. Bureaucracy was stated as the main deterrent by companies involved in both pharmaceuticals and medical equipment/biotechnology and by companies only involved in medical equipment/biotechnology, as opposed to pharmaceutical companies (Table 4). As far as the legal status of companies is concerned, it seems that multinational companies reported the corporate advertising as the most important encouraging factor to implement CSR actions than national ones for which it is moderately important. National companies stated that the lack of government incentives prevent the implementation of CSR, while multinational ones reported it as less important (Table 4).

**Table 4:** Field of companies’ operation and legal status comparisons with encouraging and deterrent factors for implementing corporate social responsibility (CSR) actions 1 = very important; 4 = less important

	Field of companies’ operation						Company’s legal status			
	Pharmaceuticals	Medical equipment/ Biotechnology products	Companies specialising in both categories				National Companies	Multinational Companies in Greece		
	Mean (SD)	Mean (SD)	Mean (SD)		P value		Mean (SD)	Mean (SD)	P value	
Encouraging factors										
Social acceptance	1.60 (0.89)	2.33 (1.13)	2.00 (1.03)		0.034					
Industry competition/ comparative advantage	2.47 (0.90)	1.75 (0.61)	2.40 (0.82)		0.003					
Corporate advertising	1.67 (0.88)	2.42 (1.14)	2.30 (0.92)		0.014		2.43 (1.07)	1.87 (0.96)	0.023	
Direct/indirect economic benefits							3.29 (0.71)	2.65 (1.06)	0.007	
Deterrent factors										
Bureaucracy	2.40 (1.16)	1.67 (0.96)	1.60 (0.94)		0.011					
lack of government incentives	2.40 (1.28)	1.67 (0.76)	2.00 (0.92)		0.040		1.64 (0.91)	2.30 (1.09)	0.009	
Financial costs							1.86 (0.65)	2.26 (0.85)	0.035	

Finally, it is worth mentioning that the vast majority (97.3%) of respondents reported that the State should reward socially responsible companies, while an equally higher percentage (91.1%) stated that there should be financial, tax incentives and rewards for companies that implement CSR actions.

## Discussion

To our knowledge, the present study is a first attempt to examine the factors affecting the implementation of CSR activities in the health technology industry in Greece. According to the study results, the majority of companies implement CSR actions and incorporate them into their business strategies. However, only three out of 10 companies seem to adhere to the international standards of the GRI. Additionally, the companies dispose adequate amounts from their annual turnover to CSR actions. Regarding the CSR encouraging factors, our results showed that the contribution to society and the ethical commitment of the company emerged as very important encouraging factors. Adversely, bureaucracy and the lack of governmental incentives’ supply are the most important obstacles. Statistically significant differences regarding the factors influencing CSR actions were observed according to the companies’ field of operation and the legal status. Nine out of 10 respondents believe that the government should reward socially responsible companies.

Our findings are in accordance with the international literature. With regard to the implementation of CSR actions at the local level, studies report that over the years, companies become more active, effective and adept in their business strategy CSR activities [37-39]. Moreover, our study findings showed a low number of companies to adhere the GRI guidelines and evaluate CSR activities. This finding is also in accordance with the study of Metaxas et al. (2013), which reported that only 41% of the companies adhere to the GRI and other international standardization systems’ guidelines [38]. Additionally, a Greek study that investigated the amount of funds that companies spend on CSR actions showed that 64% dispose up to €200 thousand [40] which is almost similar to our results. This is an important finding since the majority of companies in Greece devoted limited resources due to the recent crises, the lack of knowledge about the importance of performing CSR actions and incorporating them into their culture [41,42].

Furthermore, our findings on the factors influencing the implementation of CSR actions are in accordance with other relevant studies. Ethical corporate culture, contribution to society and improvement of corporate image motivate companies to uptake CSR actions. Findings of international studies are in agreement with our results, showing that the contribution to society and the ethical commitment of the company are the most important encouraging factors [10,11,18,43-47]. Regarding the deterrent factors of CSR, the studies of Chkanikova and Mont and El-Bassiouny [48,49] converge that the bureaucracy and the lack of government incentives are among the major obstacles. Adversely, several studies highlight the financial costs and the company’s culture as important inhibiting factors in adopting responsible practices [12-14,18,50].

Significant differences regarding the factors influencing CSR actions were observed according to the companies’ field of operation. More specifically, our finding on the factors driving CSR in pharmaceutical companies is consistent with the study by Droppert and Bennett (2015), in which reputation benefits and long-term financial returns are the most encouraging factors [21]. As far as the differences found regarding the encouraging and discouraging factors for CSR in the medical equipment and biotechnology companies, this may be due to variations related to their size, field of operation, high competition and decision-making process [10,20,51-58].

As far as the encouraging and deterrent factors for CSR compared to the company’s legal status, our results showed that multinational companies cited corporate advertising as the main encouraging factor. However, the lack of government incentives was reported by the national companies as the main deterrent. This is also an important finding, which could be explained by the fact that multinational companies, as opposed to national ones, have stronger economic foundations, different managerial cultures and, therefore, the lack of government incentives does not strongly influence their CSR decisions.

Finally, the vast majority of companies stated that the government should reward and provide incentives to socially sensitive companies. This is also a common finding reported by the studies of ICAP (2021) [40] and Pouliopoulos et al. (2012) [34], conducted in Greece, which demonstrated that the economic benefits and tax breaks could enhance CSR practices. The need for reward and incentives to socially responsible companies by the government is also stated by the Keinert (2008) study [34,59].

Study limitations 

Potential limitations of this study are, first, the lack of a standardized questionnaire should be noted as a limitation. However, the development of the specific tool combines the international with the Greek experience in the implementation of CSR actions and has been elsewhere used. Second, the diversity observed in the results regarding the implementation of CSR between national and multinational companies may reflect the organizational and ethical culture of the parent company and not the Greek entrepreneurial mentality. Third, subjective and biased responses, characteristic of all questionnaire-based research, should be considered as another limitation of the study. Finally, further research is needed in the country for obtaining more transparent and comparative results.

## Conclusions

In Greece, the majority of the health technology industry implements CSR initiatives, despite the previous economic and pandemic crises. The company's contribution to society and its ethical commitment was found to be the most important CSR drivers, while bureaucracy and the lack of government incentives are revealed as the major deterrents. The value prioritization of all CSR factors seems to be influenced by the company’s field of operation and legal status. The government rewarding socially sensitive companies would provide significant entrepreneurial and societal benefits, supporting the overall Greek economy.
